# Archetypal Analysis of Injury in Kidney Transplant Biopsies Identifies Two Classes of Early AKI

**DOI:** 10.3389/fmed.2022.817324

**Published:** 2022-04-07

**Authors:** Philip F. Halloran, Georg A. Böhmig, Jonathan Bromberg, Gunilla Einecke, Farsad A. Eskandary, Gaurav Gupta, Marek Myslak, Ondrej Viklicky, Agnieszka Perkowska-Ptasinska, Katelynn S. Madill-Thomsen, The INTERCOMEX Investigators

**Affiliations:** ^1^Alberta Transplant Applied Genomics Centre, Edmonton, AB, Canada; ^2^Division of Nephrology and Transplant Immunology, Department of Medicine, University of Alberta, Edmonton, AB, Canada; ^3^Division of Nephrology and Dialysis, Department of Medicine III, Medical University of Vienna, Vienna, Austria; ^4^Department of Surgery, University of Maryland, Baltimore, MD, United States; ^5^Department of Nephrology, Hannover Medical School, Hannover, Germany; ^6^Division of Nephrology, Virginia Commonwealth University, Richmond, VA, United States; ^7^Department of Clinical Interventions, Department of Nephrology and Kidney Transplantation Samodzielny Publiczny Wojewódzki Szpital Zespolony (SPWSZ) Hospital, Pomeranian Medical University, Szczecin, Poland; ^8^Department of Nephrology and Transplant Center, Institute for Clinical and Experimental Medicine, Prague, Czechia; ^9^Department of Transplantation Medicine, Nephrology and Internal Diseases, Medical University of Warsaw, Warsaw, Poland

**Keywords:** biopsy, kidney transplantation, injury, archetypes, gene expression

## Abstract

All transplanted kidneys are subjected to some degree of injury as a result of the donation-implantation process and various post-transplant stresses such as rejection. Because transplants are frequently biopsied, they present an opportunity to explore the full spectrum of kidney response-to-wounding from all causes. Defining parenchymal damage in transplanted organs is important for clinical management because it determines function and survival. In this study, we classified the scenarios associated with parenchymal injury in genome-wide microarray results from 1,526 kidney transplant indication biopsies collected during the INTERCOMEX study. We defined injury groups by using archetypal analysis (AA) of scores for gene sets and classifiers previously identified in various injury states. Six groups and their characteristics were defined in this population: No injury, minor injury, two classes of acute kidney injury (“AKI,” AKI1, and AKI2), chronic kidney disease (CKD), and CKD combined with AKI. We compared the two classes of AKI, namely, AKI1 and AKI2. AKI1 had a poor function and increased parenchymal dedifferentiation but minimal response-to-injury and inflammation, instead having increased expression of PARD3, a gene previously characterized as being related to epithelial polarity and adherens junctions. In contrast, AKI2 had a poor function and increased response-to-injury, significant inflammation, and increased macrophage activity. In random forest analysis, the most important predictors of function (estimated glomerular filtration rate) and graft loss were injury-based molecular scores, not rejection scores. AKI1 and AKI2 differed in 3-year graft survival, with better survival in the AKI2 group. Thus, injury archetype analysis of injury-induced gene expression shows new heterogeneity in kidney response-to-wounding, revealing AKI1, a class of early transplants with a poor function but minimal inflammation or response to injury, a deviant response characterized as PC3, and an increased risk of failure. Given the relationship between parenchymal injury and kidney survival, further characterization of the injury phenotypes in kidney transplants will be important for an improved understanding that could have implications for understanding native kidney diseases (ClinicalTrials.gov #NCT01299168).

## Introduction

Injury is universal in kidney transplants because of donation-implantation, presenting an opportunity to study the molecular characteristics associated with parenchymal damage. It is usually classified as acute kidney injury (AKI) or chronic kidney disease (CKD), but injury at the molecular level covers a wide spectrum of phenotypes. The emergence of the Molecular Microscope® Diagnostic System (1–5) for identifying rejection allows us to focus on understanding the injury component of gene expression independent of rejection. We recently analyzed injury-related features of kidney transplant biopsies as a spectrum, rather than dichotomizing between AKI and CKD (6). We performed principal component analysis (PCA) on microarray results from 1,526 indication biopsies, based on their expression of transcript sets and classifier scores associated with AKI- or CKD-related histology features, depressed estimated glomerular filtration rate (eGFR), and proteinuria. The resulting PC1 reflected no injury vs. injury, while PC2 reflected early AKI vs. late CKD. PC3 distinguished inflamed injury, including T cell-mediated rejection (TCMR; negative PC3) from uninflamed injury (positive PC3). High PC3 was increased in early AKI and CKD, and correlated with increased expression of an epithelial polarity and adherens junctions gene, PARD3, that is increased in AKI and CKD but decreased in TCMR.

This study aimed to describe the clinical classes of biopsies corresponding to these injury PC scores and understand the functional status and risk of progression, particularly in kidneys with no rejection. We used archetypal analysis (AA) to develop a classification of biopsies based on parenchymal injury features previously used for PCA (6). We studied genome-wide transcript expression measured by microarrays from 1,526 indication kidney transplant biopsies from the INTERCOMEX study (ClinicalTrials.gov #NCT01299168), following AA strategies previously used to categorize rejection-related phenotypes (3). We examined the relationships between injury archetype groups and time post-transplant, eGFR, proteinuria, rejection, histology lesions, and graft survival. Having previously defined each biopsy in terms of its molecular rejection status, our goal was to understand each kidney in terms of its parenchymal integrity and injury-induced phenotype—its response-to-wounding. This would allow all biopsies to be described both in terms of their rejection state and their injury state, and would give a complete molecular phenotype that relates to prognosis.

## Materials and Methods

The population and some methods were previously published (6).

### Statistics

All analyses were done using the R programming language (7). Because classifier and archetype scores are frequently skewed, non-parametric tests were used where applicable, for example, Spearman's test for correlations and Wilcoxon's signed-rank test for comparing medians.

### Study Population

As published (6), the 1,526 biopsies for clinical indications included in this study were obtained prospectively from established international centers (listed in Supplementary Table 1) with consent under local Institutional Review Board (IRB)-approved protocols (ClinicalTrials.gov NCT01299168). A portion (mean 3 mm) of one core was immediately stabilized in RNA*later*® and shipped to the Alberta Transplant Applied Genomics Centre (http://atagc.med.ualberta.ca) for processing. Gene expression was measured on Affymetrix PrimeView arrays unless the biopsy was inadequate for analysis [e.g., too small or RNA degraded: ~4% of biopsies (5)]; 1,745 biopsies had enough RNA quality to run on microarray chips. Of these, we used 1,679 that had been assigned histological diagnoses.

Previous analyses (8) have indicated that biopsies with high medulla content sometimes have slightly altered molecular characteristics. For this reason, we removed the 153 biopsies we estimated to have <10% cortex [by measuring expression of the glomerulus-specific gene podocin (8)], leaving 1,526 biopsies for all analyses shown in this study. No additional inclusion/exclusion criteria were used. CEL files are available on the Gene Expression Omnibus website (GSE124203).

Demographics and histological findings have been described previously for the set of 1,679 biopsies (2). Demographics of the 1,526 indication biopsies from 1,280 patients are shown in Supplementary Table 2 and histology and DSA in Supplementary Table 3.

### Histology/Clinical Data

Proteinuria and delayed graft function (DGF) were defined as per the centers' standard-of-care, as were histological diagnoses following Banff guidelines (9, 10). Since there is no “AKI” category defined by histology, we took all biopsies not diagnosed with any specific disease or condition (“no major abnormalities”) and classified them as “clinical AKI” if ≤6 weeks post-transplant, or “normal” if >6 weeks post-transplant.

### Inputs for Injury AA

The molecular injury scores used as inputs have been published (6). Further details of the pathogenesis-based transcript sets (PBTs) for U219 arrays are provided at https://www.ualberta.ca/medicine/institutes-centres-groups/atagc/research/gene-lists.

Four classifiers were used to generate input scores for AA: ci>1_Prob_ (ci-lesion score > 1 vs. ≤ 1) (11); ct>1_Prob_ (ct-lesion score > 1 vs. ≤ 1) (11); lowGFR_Prob_ (eGFR ≤30 vs. >30) (12); and Prot_Prob_ (proteinuria positive vs. negative) (12). In all cases, 12 different classifier methods were trained [see (2) for details], and the median test set scores from the 12 used as the final estimate. All scores were based on the left-out sets in 10-fold cross-validation, that is, all scores were predicted from training set models that had no information whatsoever concerning the left-out test sets they were predicting. Our previous publication (3) used the same algorithmic methods but was based on the full set of 1,679 biopsies. For reasons explained above, all classifiers were rerun using the smaller 1,526 population for this study. All classifiers were implemented with functions from the R “caret” library (13).

We used eight PBTs as input and to interpret the results: six increased in injury, namely, damage-associated molecular pattern transcripts (DAMPs) (14, 15), AKI transcripts (IRRATs) (16), injury-repair-induced transcripts day 3 (IRITD3s) and injury-repair-induced transcripts day 5 (IRITD5s) (17), immunoglobulin transcripts (IGTs) (18), and mast cell transcripts (MCATs) (19); plus two parenchymal transcript sets characteristic of well-differentiated kidney tissue, namely, KT1 (which exclude solute carriers) and KT2 (solute carriers) (20) that are decreased in injury. A PBT score is calculated as the geometric mean of the fold change of all probe sets in the PBT vs. the mean expression of those probe sets in a defined control population, that is, the mean fold change across all probe sets. We use four nephrectomy samples as our controls.

### Injury AA

Our use of AA for rejection has been published (3). AA (21) finds a small number of hypothetical archetypes that represent extreme “phenotypes” within a data set. The number of archetype clusters chosen is largely subjective. We examined models using between two and seven clusters and chose six clusters based on what we believe produced the most informative and interpretable categorization. Each sample is assigned scores for each of the six clusters, which sum to 1.0. By convention, each sample is assigned to a group (“cluster”) based on the highest of its scores.

### Visualization of Archetype Cluster Distributions

The output from the AA was six archetype scores for each of the 1,526 biopsies. This cannot easily be visualized without dimensionality reduction. It is conventional to use PCA to assign the biopsies in two- or three-dimensional space, and then color the biopsy symbols using the archetype cluster assignment [e.g., (22, 23)]. We follow this convention using the same 1,526 × 12 data matrix as input for both the PCA and AA.

### Moving-Average Plots

For all plots showing moving averages, the data were first sorted by ascending order of the x-axis variable, for example, time post-transplant. The mean of the x and y variables of the (ordered) biopsies 1-400 were then calculated and plotted. The sliding window was then incremented to biopsies 2-401, the means recalculated and plotted, and so on. Generally, these data had a great deal of scatter, and the sliding window approach was used to see general trends in the data, as are regression lines in standard linear regression.

### Survival Analysis

Three-year post-biopsy survival was analyzed using one randomly selected biopsy per patient. We used random forests (RFs), as implemented in the “randomForestSRC” package (24). RF is less sensitive to multicollinearity problems than is Cox regression and is also able to model interaction effects between predictors to some extent. Ten thousand trees were grown for each analysis using the nsplit = 1 parameter.

## Results

### Injury AA

**Figure 1:**
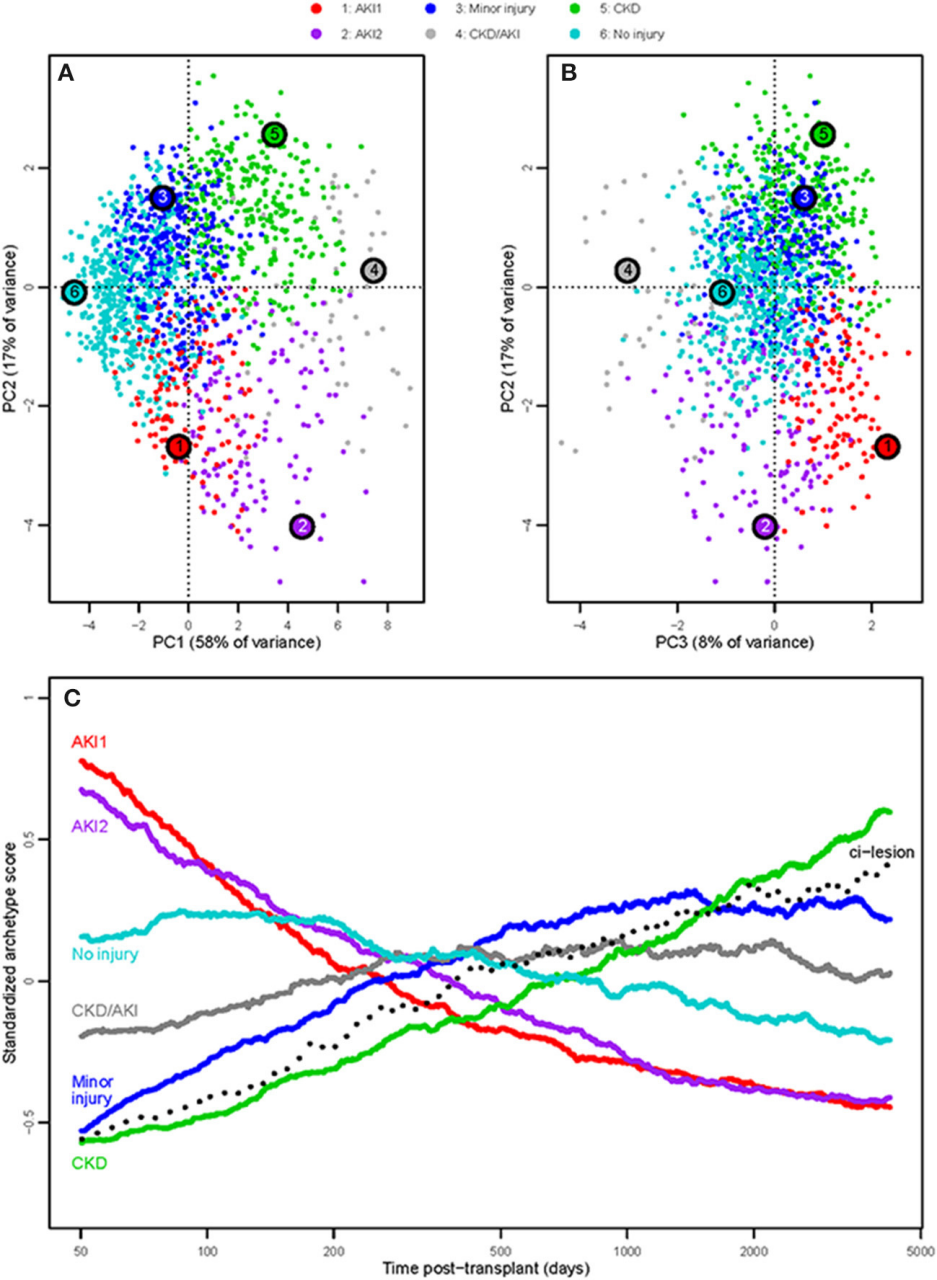
Injury-based PCA, colored by injury archetype cluster. **(A)** PC2 vs. PC1 scores for each biopsy; **(B)** PC2 vs. PC3 scores; **(C)** moving averages of standardized injury archetype scores (window size = 400 biopsies). As there are large differences in mean scores between archetypes, all scores were standardized to a mean of 0.0 before plotting. The y-axis is in standard deviation units. Biopsies sorted by ascending time of biopsy post-transplant.

Figure 1 shows the PCA distribution of biopsies described previously (6): Figure 1A, PC2 vs. 1 and Figure 1B, PC2 vs. 3. PC1 represents all molecular injury vs. uninjured tissue. PC2 separates AKI (negative PC2) from CKD (positive PC2). PC3 is a new dimension that separates uninflamed injury with high expression of some unusual epithelial transcripts such as PARD3 (positive PC3) from inflamed injury (negative PC3).

In Figure 1, biopsies are colored by their membership in the six injury archetype groups. As described below, these are completely different from the rejection archetype groups (3). The large symbols numbered 1–6 indicate the location of the actual archetype (theoretical idealized phenotype). Assignment of biopsies to archetype groups should be thought of as the “most likely” cluster based on their location in multivariate molecular space, rather than as a definitive classification system. As such, the injury archetypes are interpretable as “clinical scenarios.” Based on the results outlined below, we assigned provisional descriptive labels based on clinical and molecular characteristics to each of the six injury clusters, namely, no injury, minor injury, AKI1, AKI2, CKD, and CKD/AKI. We introduced the names here for clarity.

Notably, the AKI1 differs from AKI2 in all three PC dimensions, namely, less PC1, is shifted more positively in PC2, but strongly positive PC3.

### Distribution of the Injury AA Scores Over Time Post-transplant

Figure 1C shows the moving averages of the six archetype scores vs. time post-transplant.

The AKI1 and AKI2 scores were highest immediately post-transplant then declined steadily. CKD scores rose steadily, similar to the rise in histological atrophy-scoring scores reflecting the cumulative burden of injury (donation-implantation and later injuries). The minor injury score rose with time, somewhat like CKD, but plateaued late. Histological fibrosis, shown here as a dotted line, rose steadily with time as previously reported, paralleling the CKD score (4).

The no injury score peaked at 3–7 months (as AKI changes recovered), then declined slowly over time as expected in an indication biopsy population as CKD increased.

### Clinical Features of the Injury AA Groups

The clinical characteristics of the six archetype groups are summarized in Table 1. The no injury group is the most normal (least amount of injury) and can serve as a control.

**Table 1:** Summary of clinical features of the injury archetype groups.

		**AKI1**	**AKI2**	**Minor injury**	**CKD/AKI**	**CKD**	**No injury**
	***N***	127	130	377	55	310	527
Clinical variables for all biopsies	Median time of biopsy post-transplant (mean)	***40 (181)***	51 (157)	1,070 (1,875)	867 (1,670)	**2,102 (2,674)**	365 (1,088)
	Mean eGFR (cc/min/m^2^)	***22***	27	52	32	35	**53**
	Mean donor age (median)	**50 (54)**	46 (48)	***41 (42)***	46 (48)	45 (46)	43 (44)
	% of column with donor age >50 years	**63%**	43%	***32%***	45%	41%	33%
	Deceased donor (%)	**89%** ^a^	65%	67%	73%	73%	***60%***
Clinical variables for biopsies with no molecular rejection	Median time of biopsy post-transplant (mean)	32 (177)	***19 (81)***	1,050 (1,929)	1,221 (1,412)	**2,510 (2,890)**	309 (1,089)
	Mean eGFR (cc/min/m^2^)	***21***	26	**54**	48	35	52
	Mean donor age (median)	**50 (54)**	50 (52)	***43 (43)***	44 (40)	47 (50)	44 (45)
	% of column with donor age >50 years	**62%**	59%	***33%***	40%	51%	36%
	Deceased donor (%)	89%^b^	74%	70%	**100%**	75%	***59%***

*The highest in each row is bolded and shaded. The lowest in each row is bolded and italicized*.

a *Fisher's exact test between AKI1 vs. AKI2 p < 0.001*.

b *Fisher's exact test between AKI1 vs. AKI2 p = 0.02*.

Groups AKI1 (*N* = 127) and AKI2 (*N* = 130) were both relatively early (median 40 and 51 days post-transplant). The CKD group (*N* = 310) was the latest post-transplant (2,102 days). The no injury group was earlier on average than minor injury (365 vs. 1,070 days).

Both AKI1 and AKI2 had low eGFR (22 and 27, respectively). The small CKD/AKI group (*N* = 55) had a low mean eGFR (32). The no injury group (*N* = 527) had a relatively normal eGFR (53), as did the minor injury group (52).

The lowest donor age and % donors >50 was in the no injury and minor injury groups. More AKI1 biopsies were from donors >50 years of age (63 vs. 43%), but this difference disappeared when only the biopsies without rejection were considered; both groups had many kidneys from older donors. Aging may be operating in both AKI groups as expected; older donor kidney results in more early dysfunction.

The AKI1 group had the most deceased donors and the no injury group had the least.

AKI1 was also strongly associated with deceased donors compared to AKI2 and the other groups, particularly the no injury group.

Thus, injury groups assigned exclusively by the molecules and machine learning have strong clinical associations. Most of these results were similar when all rejection was excluded and are thus relatively independent of the rejection states of these biopsies.

### Transcript Set Scores in the Injury AA Groups

In Table 2, the injury archetype groups AKI1 and AKI2 had many of the features expected in molecular AKI compared to the no injury, such as recent injury transcripts, dedifferentiation, macrophage transcripts, and injury PC1 (the degree of global injury). However, AKI1 differed markedly in many scores from AKI2.

**Table 2:** Mean scores for AKI and CKD-related pathogenesis-based transcript sets (PBTs) in injury archetype groups (*N* = 1,526).

**Biological processes**	**Mean transcript set and classifier score^a^ in biopsies grouped by highest archetype score**	**AKI1**	**AKI2**	**Minor injury**	**CKD/AKI**	**CKD**	**No injury**
		**(*N* = 127)**	**(*N* = 130)**	**(*N* = 377)**	**(*N* = 55)**	**(*N* = 310)**	**(*N* = 527)**
PBTs increased by recent injury	AKI transcripts (IRRATs)	1.70	**2.43** ^c^	1.16	2.26	1.72	***0.95***
	IRITD3	1.09	1.20^c^	1.02	**1.21**	1.12	***0.96***
	IRITD5	1.28	1.58^c^	1.26	**1.72**	1.42	***1.15***
PBTs increased in atrophy-fibrosis	IGTs	***0.87***	1.37^c^	2.59	**6.47**	3.56	1.38
	MCATs	***1.10***	1.47^c^	2.80	**5.36**	5.26	1.46
	BATs^b^	***1.03***	1.17^c^	1.16	**1.67**	1.27	1.07
Parenchymal transcript PBTs decreased by injury	KT1	0.85^c^	0.68	0.90	***0.47***	0.78	**0.93**
Macrophage infiltration PBTs	QCMATs^b^	1.26	2.24^c^	1.40	**2.44**	1.50	***1.19***
	AMATs^b^	1.37	2.43^c^	1.52	**2.69**	1.74	***1.24***
Injury PC1		−0.07	2.6^c^	−0.61	**5.68**	2.47	***−2.26***
Injury PC2		*−1.81*	**−2.35** ^c^	0.56	−0.21	**1.29**	−0.12
Injury PC3		**1.26** ^c^	−0.39	0.21	***−1.98***	0.49	−0.44

*The highest in each row is bolded and shaded. The lowest in each row is bolded and italicized*.

a *The gene sets were derived in human cell lines, human transplants, and mouse models to reflect biological processes relevant to rejection and injury*.

b *These were the transcript sets or classifiers not used in the Injury AA analysis*.

c *t-test of AKI1 vs. AKI2 p < 0.001*.

*AMAT, alternative macrophage associated transcripts 1; BATs, B cell-associated transcripts; DAMPs, damage-associated molecular pattern transcripts; IGTs, immunoglobulin transcripts; IRITD3, injury-repair-induced transcripts day 3; IRITD5, injury-repair-induced transcripts day 5; IRRAT, AKI transcripts; KT1, kidney parenchymal transcripts 1; KT2, kidney parenchymal transcripts 2; MCATs, mast cell transcripts; QCMAT, quantitative constitutive macrophage-associated transcripts*.

Compared to no injury, the AKI1 and AKI2 groups had increased expression of recent injury-induced transcript sets, but AKI1 was always lower than AKI2.

Transcript sets increased in atrophy-fibrosis (reflecting plasma cell, mast cell, and B cell infiltration) were very low in AKI1 compared to AKI2 and all other groups, even the no injury group.

Dedifferentiation, loss of normal kidney parenchymal transcripts (KT1), was less in AK1 than in AKI2.

Macrophage transcripts were increased in AKI1 and AKI2 but were less in AKI1 than AKI2.

Compared to AKI2, AKI1 had lower injury PC1 scores and less negative injury PC2 scores, indicating less response-to-wounding in AKI1 than AKI2. But AKI1 had a strong positive injury PC3 score, whereas AKI2 had a negative PC3 score, indicating a deviant response in AKI1 vs. AKI2.

AKI1 biopsies did have injury-induced features when compared to no rejection biopsies, specifically a response-to-wounding. However, these injury features in AKI2 were more distinct.

The CKD and CKD/AKI groups had elevated expression of a recent injury and atrophy-fibrosis-related transcripts, and CKD/AKI had the highest expression of macrophage transcripts and the greatest loss of parenchymal transcripts (KT1). PC1 was highest in CKD/AKI and PC2 was highest in CKD.

These results were similar when all rejection was excluded (Supplementary Table 4).

### Molecular Rejection in the Injury Archetype Groups

AKI1 had the fewest molecular TCMR diagnoses of any group (1/127, <1%), even less than in the no injury group (Table 3). Only 14% of AKI1 had any rejection, mostly early-stage antibody-mediated rejection (EABMR). In contrast, the AKI2 biopsies had 39% TCMR and 57% rejection overall. The small group 4 CKD/AKI (*N* = 55) had 87% rejection. Rejection was present in about half of minor injury and CKD biopsies, and 18% of biopsies with no injury, mostly EABMR.

**Table 3:** Distribution of molecular rejection diagnoses in injury archetype groups.

**Rejection archetype groups**	**Number of biopsies in each injury archetype group (% of column total)**						
	**AKI1**	**AKI2**	**Minor**	**CKD/AKI**	**CKD**	**No-injury**	**Total**
Early-stage ABMR (EABMR)	12	11	73	*1*	39	49	185
Fully-developed ABMR (FABMR)	3	*5*	76	6	43	17	150
Late-stage ABMR (LABMR)	2	7	23	5	30	*8*	*75*
TCMR (TCMR1+TCMR2)	*1*	51	*20*	36	40	23	171
*% of column with rejection^a^ (number)*	***14%*** **(18)**	57% (74)	51% (192)	**87% (48)**	49% (152)	18% (97)	38% (581)
*% of column with no rejection^a^ (number)*	**84% (109)**	43% (56)	49% (185)	***13%*** **(7)**	49% (158)	82% (430)	62% (945)
**Total**	127	130	377	55	310	527	1,526

a *The highest % in these rows is bolded and shaded. The lowest is bolded and italicized. The lowest is bolded and italicized*.

Histological diagnoses in the injury archetype groups are presented in Table 4. In general, the pattern was in agreement with the molecular rejection groups. There were few diagnoses of rejection in the AKI1 group, with most AKI1 assessed as relatively normal or mild atrophy-fibrosis [this is consistent with our previous finding that molecular AKI changes that correlate with eGFR loss are not consistently detectable by histology (16)].

**Table 4:** Distribution of histological diagnoses in the injury archetype groups (grouped by highest injury score) (*N* = 1,526).

**Histology diagnosis (*****N*** **=** **1,526)**			**# of biopsies in each injury archetype group (%of column total)**						**Total**
			**AKI1**	**AKI2**	**Minor**	**CKD/AKI**	**CKD**	**No-injury**	
**Total**			127	130	377	55	310	527	1,526
# Rejection *N* = 708 (36%)	**ABMR-related**	ABMR	11	14	**114**	***7***	78	65	289
		Transplant Glomerulopathy (TG)	***0***	***0***	**21**	2	17	6	46
		ABMR suspected	2	2	**10**	***1***	7	7	29
	**Mixed**		***0***	6	**18**	10	15	6	55
	**TCMR-related**	TCMR	***7***	**32**	21	13	19	**32**	124
		BK	***1***	10	6	6	8	**14**	45
		**Borderline**	10	14	23	***3***	17	**53**	120
	All rejection-related (% of column)		***31 (24%)***	78 (60%)	**213 (56%)**	42 (76%)	161 (52%)	183 (35%)	708 (46%)
# No rejection *N* = 818 (64%)	No major histologic abnormalities (“Normal”)		65	30	73	***3***	41	233	**445**
	Diabetic Nephropathy		1	***0***	7	***0***	**11**	4	23
	Glomerulonephritis		3	***1***	**37**	***1***	27	**37**	106
	IFTA-no other disease		18	12	36	***8***	**52**	49	175
	Other		9	9	11	**1**	18	**21**	69
	All with no rejection (% of column)		96 (76%)	52 (40%)	164 (44%)	***13 (24%)***	149 (48%)	**344** (**65%**)	818 (54%)
Histology scores	Interstitial fibrosis (mean ci)		0.97	0.90	1.26	1.90	**2.01**	***0.81***	1.24
	Tubular atrophy (mean ct)		0.82	0.69	1.13	1.78	**1.84**	***0.77***	1.12

*The highest value per row is bold with shading; the lowest is bolded and italicized*.

### Relationship of Injury and Rejection AA Scores to eGFR

**Figure 2:**
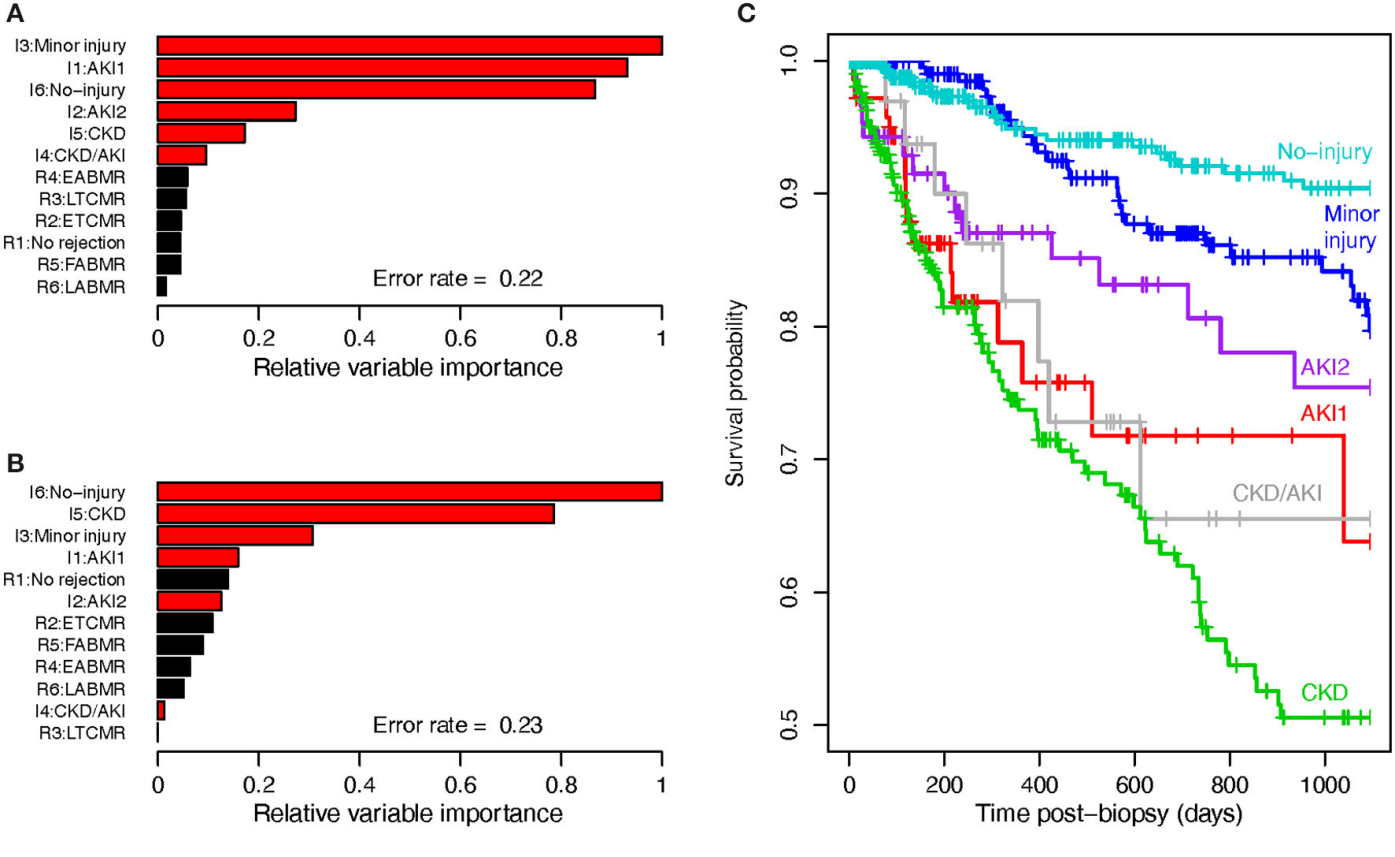
Graft survival analyses. **(A)** Random forests assessing variable importance in predicting low eGFR (eGFR < 30). **(B)** Random forests assessing variable importance in predicting 3-year death-censored graft survival. **(C)** Kaplan-Meier curves showing 3-year post-biopsy actuarial survival in the six AA injury archetype groups.

In Figure 2A, we used RFs to examine the relative importance of molecular injury archetype scores and rejection archetype scores (3) for predicting disturbed function (eGFR ≤ 30). Injury scores were strongly predictive of poor function, while rejection scores were not.

We also used random forests to assess the relative importance of molecular rejection and injury AA scores in terms of predicting graft failure within 3 years of biopsy (Figure 2B). Injury scores were more important than rejection scores alone (error rate 0.35, data not shown). These findings using injury archetypes are consistent with our previous analyses using other injury measurements (12).

The actuarial survival curves for the six injury archetype groups are shown in Figure 2C (3-year death censored graft survival after biopsy). The poorest survival was in CKD and CKD/AKI, but survival was also poor in AKI1, even though this group had less rejection. The best survival was in the no injury followed by the minor injury group.

### Analysis of Early (≤6 Weeks) Non-rejecting Biopsies

We selected all early biopsies ≤6 weeks post-transplant that had also been designated “no rejection” by the rejection archetype model (3) (*N* = 171 of 201 early biopsies shown in Table 5), permitting us to study a pure set of early biopsies with no rejection.

**Table 5:** Distinct phenotype in early biopsies (≤6 weeks post-transplant) with no molecular rejection^a^ (*N* = 171).

**Variable**		**Injury archetype**			
**Mean scores in:**		**AKI1 (*****N*** **=** **58)**	**AKI2 (*****N*** **=** **41)**	**No-injury (*****N*** **=** **64)**	**Others (*****N*** **=** **8)**^**d**^
Time of biopsy post-transplant		**17**	**16**	21	28
Fraction donors >50 years of age		**28/48 (58%)**	**20/36 (56%)**	*25/59 (42%)*	5/7 (71%)
Fraction deceased donors (%)		**93%**	**74%**	*46%*	63%
PARD3 (top PC3 increased gene)		**268** ^**c**^	**233**	*177*	178
ANXA2 (top PC1 increased gene)		**1,074** ^**b**^	**1,551**	*810*	1,154
PBTs increased by recent injury	IRRAT	**1.79** ^**b**^	**2.63**	*1.05*	1.52
	IRITD3	**1.10** ^**b**^	**1.24**	*0.98*	1.11
	IRITD5	**1.26** ^**b**^	**1.56**	*1.21*	1.48
PBTs decreased in injury	KT1	**0.85** ^**b**^	**0.71**	0.94	0.81
Histology lesions	Interstitial fibrosis (Banff ci-score)	**0.77**	**0.58**	0.69	*0.25*
	Interstitial infiltrate (Banff i-score)	**0.16** ^**c**^	**0.56**	0.41	*0.00*
	Total inflammation (Banff ti-score)	**0.11** ^**c**^	**0.57**	0.69	*0.00*
Renal function	Creatinine at biopsy median (mmol/L)	**431**	**405**	*165*	186
	eGFR at biopsy median (cc/min/m^2^)	**16.0**	**15.5**	45.0	45.0
Fraction failed by 3 years post biopsy (death censored)		**12/46 (26%)** ^**e**^	**3/39 (8%)**	4/59 (7%)	*0/7 (0%)*
Fraction designated delayed graft function (DGF) (%)		**31/58 (53%)**	**19/41 (46%)**	*7/64 (11%)*	1/8 (13%)

a *Defined as molecular rejection archetype 1 (no rejection)*.

b *p-value < 0.001 based on t-test between AKI1 and AKI2*.

c *p-value < 0.05 based on t-test between AKI1 and AKI2a*.

d *Others include minor, CKD/AKI, and CKD groups*.

e *p-value < 0.05 based on Fisher's exact test between AKI1 and AKI2*.

By injury archetype assignments, these 171 biopsies included 58 AKI1, 41 AKI2, and 64 no injury. Eight were in other injury archetype clusters.

In these biopsies, AKI1 and AKI2 were similar in mean time post-transplant, % donor age>50, renal function, and rates of DGF. AKI1 had a higher fraction of deceased donors (93 vs. 74%).

AKI had a higher expression of the top PC3 gene, PARD1; AKI2 had a higher expression of the top PC1 gene, ANXA2, and of all PBTs increased by recent injury and more loss of parenchymal transcripts.

Interstitial fibrosis was low in both (although higher in AKI1), and interstitial inflammation was less in AKI than AKI2.

The rate of graft loss by 3 years was higher in AKI1 kidneys (26%) than in AKI2 kidneys (8%).

The striking differences between early biopsies with AKI1 vs. AKI2 are that AKI1, despite a very low eGFR, has less molecular injury change, less inflammation, less parenchymal dedifferentiation but instead has increased PC3 and increased expression of the top PC3-correlated gene, PARD3.

### Relationships Between AA Injury Groups and Graft Survival in Biopsies With No Rejection

**Figure 3:**
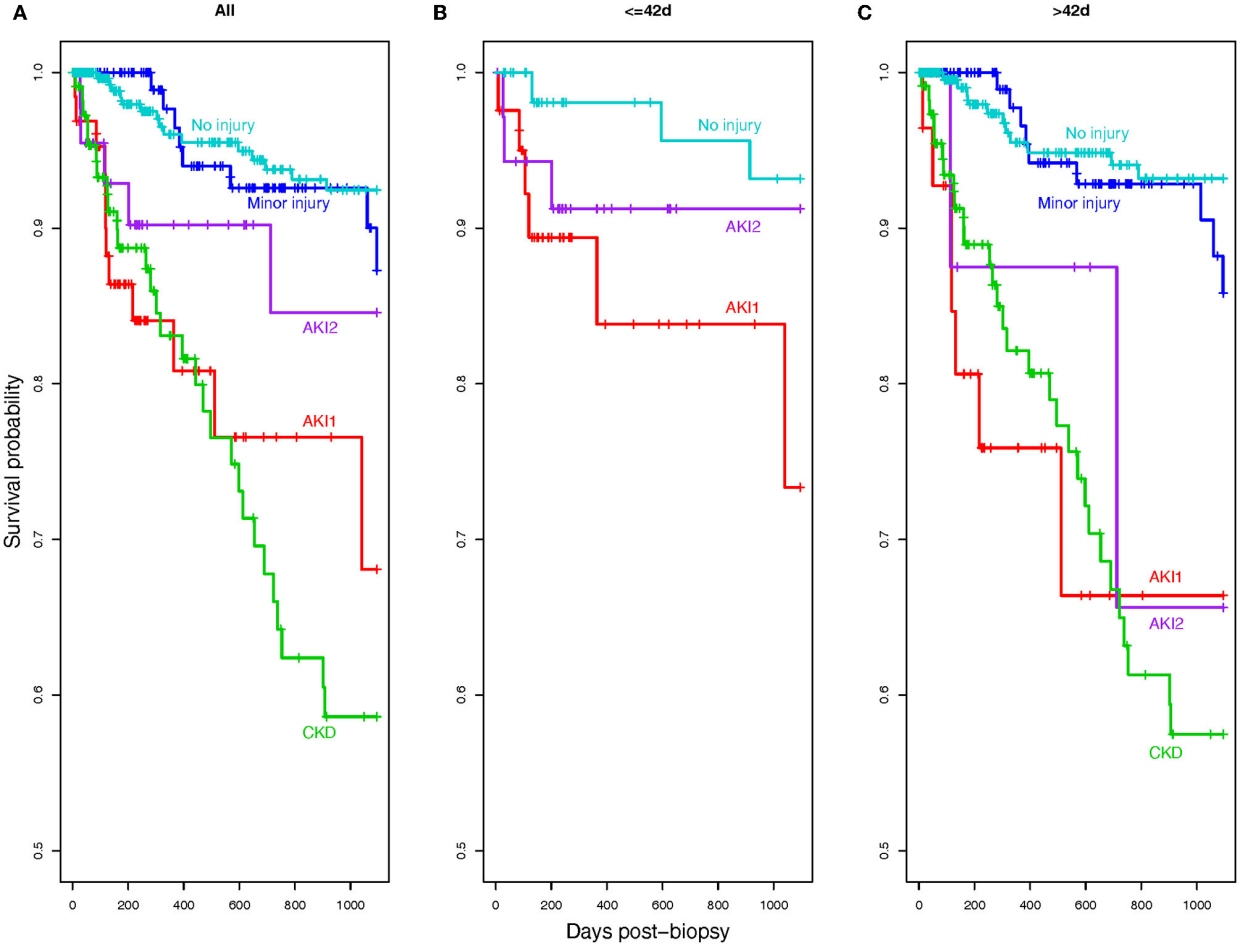
Kaplan-Meier curves showing actuarial 3-year death-censored graft survival in the injury AA groups after excluding all biopsies with rejection. **(A)** Injury AA groups were compared across time post-biopsy. We also examined the differences between, **(B)** short-term (≤42 days post-biopsy), and **(C)** long-term (>42 days post-biopsy) survival probabilities. The CKD/AKI group was removed from these plots due to the very small group size and scarcity of events.

Biopsies with no molecular rejection were grouped by AA assignment, and 3-year survival probability was assessed for each group (Figure 3) (after removing rejection, the CKD/AKI group was too small for reliable survival estimates).

In all biopsies, the CKD and AKI1 groups had the worst survival probability, while the no injury group had the best prognosis (Figure 3A).

When only biopsies within 42 days post-transplant were considered, AKI1 displayed impaired survival probabilities (Figure 3B) (there was no CKD).

When biopsies later than 42 days post-transplant were considered, AKI1, AKI2, and CKD all had reduced survival probability (Figure 3C).

Thus, AKI1 with no rejection had impaired short-term outcomes despite (or possibly because of) the relative lack of typical AKI changes and inflammation.

## Discussion

This study was designed to establish a molecular classification of parenchymal injury-related scenarios that extended beyond simply designating AKI and CKD and define the relationship of these new groups to histology, rejection, function, and survival. Having previously used molecular rejection features measured in genome-wide microarrays to classify rejection (2), we now extended this approach to describe the injury-related classes, and define both the “injury-ness” and the “rejection-ness” features of every biopsy. We explored injury scenarios in 1,526 kidney transplant indication biopsies, taken between 1 day and 33 years post-transplant. We assessed injury using 12 predefined scores, including transcripts induced in AKI and CKD, transcripts lost with kidney injury, and injury-related classifiers reflecting atrophy, fibrosis, low eGFR, and proteinuria as previously used for our injury PCA (6). AA produced six scores per biopsy, and clusters were assigned based on the highest score of the six. The six clusters included two groups with early injury, AKI1 and AKI2, with severe dysfunction and frequent DGF; CKD, CKD/AKI, minor, and no injury. AKI1 and AKI2 differed in that AKI1 had lower expression of the usual AKI-induced genes such as ANXA2, little inflammation, and virtually no TCMR. Compared to AKI2, AKI1 also had lower injury-induced transcripts (e.g., IRRATs), lower macrophage transcripts, and less parenchymal dedifferentiation. However, AKI1 had high PC3 and related gene PARD3. In other words, AKI1 had less evidence of the conventional response-to-wounding than AKI2 despite severely impaired function and instead had an alternative or deviant response, PC3. The best predictors of disturbed function (eGFR ≤ 30) and graft loss were injury archetypes, not rejection archetypes. High rates of failure occurred in CKD and AKI1 even when rejection was excluded (4). We conclude that it is important to recognize the diversity in injury classes when interpreting kidney transplant biopsies. Assessing injury phenotypes provides novel insights into changes that are largely silent in histology (16) and profoundly affect function and prognosis.

A comparison of the 127 AKI1 biopsies to the 130 AKI2 biopsies revealed previously unknown heterogeneity in early kidney transplant biopsies, particularly in those from deceased donors, and invited a specific examination of the early biopsies before 6 weeks post-transplant. Removing biopsies with rejection from the early (≤6 week) cohort showed diversity in the phenotype of early kidney transplant dysfunction independent of rejection. AKI1 with no rejection still had severe dysfunction, abundant DGF, but virtually no inflammation.

Although increased AKI1 and AKI2 scores were both common with donor age >50, we remain concerned that preexisting somatic cell senescence mechanisms could be playing a role in AKI1, processes that are not readily assessed in genome-wide biopsy studies. Aging and senescence are not necessarily predicted by calendar age, and the possibility remains that aging/senescence processes were more advanced in kidneys that developed AKI1 after donation, almost always from deceased donors. The PC3-related changes in AKI1 such as increased PARD3 remind us that AKI1 is not only deficient in the usual AKI-induced response-to-wounding but also deviates toward other PC3-related characteristics.

**Figure 4:**
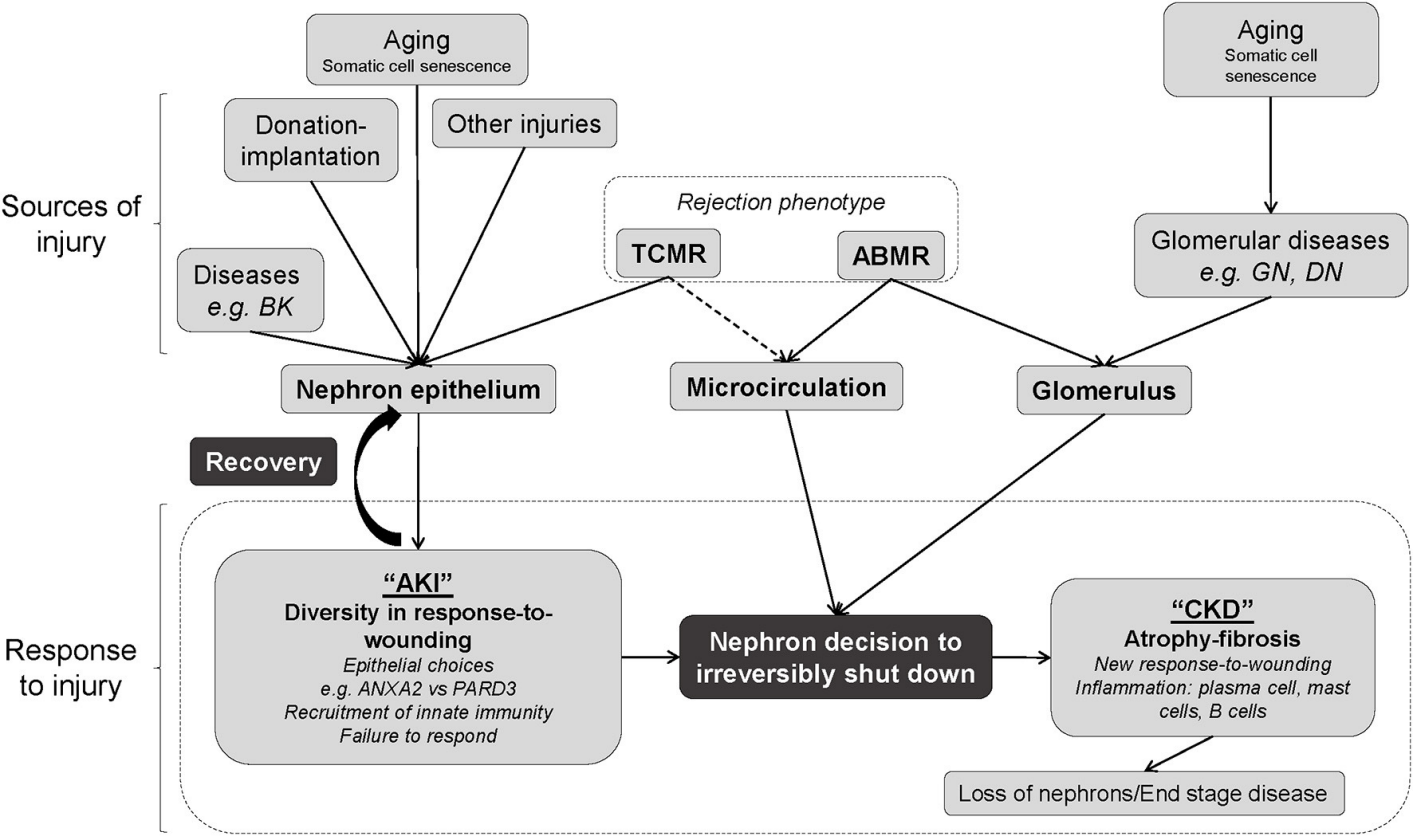
Schematic diagram representing the relationships between sources of injury and response to injury in kidney transplant biopsies based on these analyses. Interplay between sources of injury, pre-existing limitations such as aging, and response to injury by the nephron. There are two routes to irreversible nephron shutdown, namely, the epithelial injury and through glomerulus injury. Epithelial injury should trigger the response-to-wounding, which involves epithelium, matrix, and microcirculation, and evokes innate immunity. Failure to mount a response to wounding and adopting a “PC3”-related response (e.g., PARD3) with minimal inflammation leads to failure to recover. Many sources of injury (separate from and including rejection) interact with the nephron epithelium, producing acute kidney injury (AKI). In this instance, the epithelium can be repaired and the organ can recover, or progress to nephron failure. Alternatively, aging and/or ABMR can contribute to glomerular disease and ABMR can additionally affect the microcirculation, affecting the glomerulus and again causing nephron shutdown, which eventually leads to chronic kidney disease (CKD). If this occurs, a loss of nephrons and end-stage renal disease may occur. Different sources of injury may interact to cause many forms of injury, and injury itself predicts the graft survival while the rejection status does not. Thus, defining the heterogeneity within biopsy injury is an important part of clinical management.

Molecular injury measurements are critical in understanding functional disturbance and outcomes because wounding is an intermediate phenotype that integrates the total burden of parenchymal damage from donation-implantation, rejection, recurrent disease, BK, other insults, and advancing biological aging. Figure 4 represents the potential links between these sources of parenchymal injury and eventual organ failure. The injury phenotypes themselves are a final common pathway to be distinguished from the upstream injury-inducing mechanisms and diseases that are the critical targets of treatment. Perhaps treatments of the injured tissue itself will eventually emerge, possibly targeting some of the key molecules induced by injury. The sources of injury should be avoided or promptly treated if possible, but they are not always identified, and their effects may linger after apparently successful treatment, for example, after successful treatment of TCMR. Thus, injury phenotypes can be misinterpreted as autonomous when the cause of injury is either not detected or no longer operating. Moreover, badly injured tissue may also progress autonomously at some stage, for example, nephron loss may progress autonomously due to podocyte loss (25).

Rejection is a major source of parenchymal injury and has complex relationships with injury phenotypes because TCMR and ABMR have different effects on the parenchyma. TCMR is an interstitial process almost always associated with parenchymal injury. The AKI1 phenotype virtually excludes TCMR. By contrast, EABMR is usually associated with minimal injury because EABMR is a glomerular/microcirculation disease that usually has little initial impact on parenchymal function. In ABMR, nephrons do not usually drop out until the glomeruli deteriorate with double contours (FABMR), beginning the development of CKD. There are two pathways from rejection to parenchymal deterioration: direct, as in TCMR, and indirect through glomerular damage and eventual nephron shutdown, as in ABMR. The latter may be relevant to primary glomerular diseases such as diabetic nephropathy and glomerulonephritis, where nephrons are spared until the glomerular changes are advanced.

Distinguishing between AKI1 and AKI2 may be useful in the management, given that AKI2 changes seem to predict recovery better than AKI1 changes, but recognizing such heterogeneity could be particularly useful in evaluating injury prevention and treatment strategies. We anticipate that interventions directed at typical AKI-related changes that are prominent in AKI2 may be less successful in AKI1. This heterogeneity within AKI may help us to understand why treatments for AKI have met with little success, as well as distinguish those patients who are less likely to recover.

The strengths of this analysis include the large unselected study population from multiple centers with detailed phenotyping sampled over a wide range of time post-transplant. However, the restriction to indication biopsies in IRB protocols imposes limitations in that we do not know the natural history of the molecular changes in individual kidneys. To some extent, intrastudy comparisons such as AKI1 vs. AKI2, and the use of the no injury group as an internal control offset these limitations and allow us to see the natural history of the population. Also, the risk predictions in the present study exclusively use molecular features, but incorporating major clinical variables such as eGFR and proteinuria and histology atrophy-fibrosis lesions may improve risk predictions (12).

The injury-induced changes in kidney transplants (separated from rejection processes) have lessons for native kidney diseases in general, in that primary diseases drive injury but the injury phenotypes based on the parenchymal state are the final common pathway determining function and prognosis. AKI and CKD are a useful dichotomy for epidemiological analysis (26), but the molecular states are a spectrum based on continuous numbers and reveal new classes that are clinically important such as the uninflamed but high-risk AKI1 group of damaged kidneys, many from older donors. In this sense, the richness of biopsies, data, and phenotypes available in the kidney transplant population provides potentially useful insights for native kidney disease studies.

## Data Availability Statement

The datasets presented in this study can be found in online repositories. The names of the repository/repositories and accession number(s) can be found at: https://www.ncbi.nlm.nih.gov/, GSE124203.

## Ethics Statement

The studies involving human participants were reviewed and approved by Established International Centers (listed in Supplementary Table 1) with consent under local IRB-approved protocols (ClinicalTrials.gov NCT01299168). The patients/participants provided their written informed consent to participate in this study.

## Author Contributions

PH and KM-T collected, analyzed, interpreted the data, and prepared the manuscript. GB, JB, GE, FE, GG, MM, OV, and AP-P interpreted the data and critically revised the manuscript. All authors contributed to the article and approved the submitted version.

## Funding

This research has been principally supported by grants from Genome Canada, Canada Foundation for Innovation, the University of Alberta Hospital Foundation, the Alberta Ministry of Advanced Education and Technology, the Mendez National Institute of Transplantation Foundation, and Industrial Research Assistance Program. Partial support was also provided by funding from a licensing agreement with the One Lambda Division of Thermo Fisher. PH held a Canada Research Chair in Transplant Immunology until 2008 and currently holds the Muttart Chair in Clinical Immunology.

## Conflict of Interest

PH is a consultant to Natera, holds shares in Transcriptome Sciences Inc (TSI), a University of Alberta research company dedicated to developing molecular diagnostics, supported in part by a licensing agreement between TSI and Thermo Fisher, and by a research grant from Natera. The remaining authors declare that the research was conducted in the absence of any commercial or financial relationships that could be construed as a potential conflict of interest.

## Publisher's Note

All claims expressed in this article are solely those of the authors and do not necessarily represent those of their affiliated organizations, or those of the publisher, the editors and the reviewers. Any product that may be evaluated in this article, or claim that may be made by its manufacturer, is not guaranteed or endorsed by the publisher.

## Abbreviations

AA: archetypal analysis
ABMR: antibody-mediated rejection
AKI: acute kidney injury
ATAGC: Alberta Transplant Applied Genomics Centre
ci>1Prob: classifier for fibrosis
CKD: chronic kidney disease
ct>1Prob: classifier for atrophy
DGF: delayed graft function
EABMR: early-stage ABMR
eGFR: estimated GFR
FABMR: fully-developed ABMR
IGTs: immunoglobulin transcripts
IRRAT: AKI transcripts
IRITD3: injury-repair-induced transcripts day 3
IRITD5: injury-repair-induced transcripts day 5
KT1: kidney parenchymal transcripts 1
KT2: kidney parenchymal transcripts 2
LABMR: late-stage ABMR
lowGFRProb: classifier for probability of eGFR ≤ 30
MCATs: mast cell transcripts
MMDx: Molecular Microscope® Diagnostic System
PBTs: pathogenesis-based transcript set
PCA: principal component analysis
PC1: principal component 1
PC2: principal component 2
PC3: principal component 3
ProtProb: classifier for probability of proteinuria
RF: random forest
TCMR: T cell-mediated rejection
Time post-transplant: time of biopsy post-transplant.
