# Supplementary material for: Archetypal Analysis of Injury in Kidney Transplant Biopsies Identifies Two Classes of Early AKI
Source: Front Med (Lausanne). 2022 Apr 7;9:817324. doi: 10.3389/fmed.2022.817324 (PMC9021747; doi:10.3389/fmed.2022.817324)
Supplement: Supplementary file 1 [file Table_1.DOCX]

**SUPPLEMENTARY MATERIAL**

**Archetypal analysis of injury in kidney transplant biopsies identifies two classes of early AKI**

Philip F. Halloran^1,2^, Georg A. Böhmig^3^, Jonathan Bromberg^4^, Gunilla Einecke^5^, Farsad Eskandary^3^, Gaurav Gupta^6^, Marek Myslak^7^, Ondrej Viklicky^8^, Agnieszka Perkowska-Ptasinska^9^, Katelynn S. Madill-Thomsen^1^ and the INTERCOMEX Investigators^11^

^1^Alberta Transplant Applied Genomics Centre, Edmonton, Alberta, Canada; ^2^Department of Medicine, Division of Nephrology and Transplant Immunology, University of Alberta, Edmonton, Alberta, Canada;

^3^Division of Nephrology and Dialysis, Department of Medicine III, Medical University of Vienna, Vienna, Austria; ^4^University of Maryland, Baltimore, MD, USA; ^5^Department of Nephrology, Hannover Medical School, Hannover, Germany; ^6^Division of Nephrology, Virginia Commonwealth University, Richmond, VA, United States; ^7^Pomeranian Medical University, Department of Clinical Interventions, Department of Nephrology and Kidney Transplantation SPWSZ Hospital, Szczecin, Poland; ^8^Department of Nephrology and Transplant Center, Institute for Clinical and Experimental Medicine, Prague, Czech Republic; ^9^Department of Transplantation Medicine, Nephrology and Internal Diseases, Medical University of Warsaw, Warsaw, Poland; ^11^Details listed in **Table S1**.

Table of Contents

[**Table S1.** Participating Centers 3](#_Toc87964617)

[**Table S2.** Demographics in 1526 biopsies 4](#_Toc87964618)

[**Table S3.** Histologic diagnoses and HLA antibody status (All biopsies N=1526) 5](#_Toc87964619)

[**Table S4.** Mean scores for AKI and CKD-related pathogenesis-based transcript sets (PBTs) in biopsies with no molecular rejection (N=945) 6](#_Toc87964620)

| **Table S1.** Participating Centers | | |
| --- | --- | --- |
| **Center** | **Principal investigators** | **Samples contributed** |
| University of Alabama | Roslyn Mannon | 23 |
| Val d’Hebron University Hospital | Daniel Serón and Joana Sellarés | 62 |
| Montefiore Medical Center | Enver Akalin | 27 |
| Manchester Royal Infirmary | Declan de Freitas | 39 |
| University of Maryland | Jonathan Bromberg and Matt Weir | 69 |
| Charite-Medical University of Berlin | Klemens Budde | 10 |
| Medical School of Hannover | Gunilla Einecke | 59 |
| PinnacleHealth Transplant Associates | Harold Yang and Seth Narins | 11 |
| Henry Ford Hospital | Milagros Samaniego-Picota | 1 |
| Necker Hospital & St. Louis Hospital | Carmen Lefaucheur, Alexandre Loupy | 192 |
| Pomeranian Medical University | Marek Myslak | 2 |
| Warsaw Medical University | Agnieszka Perkowska-Ptasínska |  |
| Methodist Transplant and Specialty Hospital | Adam Bingaman | 76 |
| Washington University at St. Louis | Daniel Brennan and Andrew Malone | 16 |
| The Hennepin County Medical Center | Bertram Kasiske | 6 |
| University of Alberta | Philip F Halloran | 477 |
| University of Minnesota at Fairview | Arthur Matas | 72 |
| University of Wisconsin | Arjang Djamali | 8 |
| Medical University of Vienna | Georg Böhmig and Farsad Eskandary | 164 |
| Virginia Commonwealth University | Gaurav Gupta | 212 |
| **TOTAL** |  | **1526** |

| **Table S2.** Demographics in 1526 biopsies | | | |
| --- | --- | --- | --- |
| **Patient Demographics** | | **All patients**  **(N=1280)** | **No rejection cohort patients**  **(N=806)** |
| ***Mean recipient age at biopsy (range)*** | | 51 (19 – 91) | 52 (19 - 91) |
| ***Recipient Gender Male (% male)*** | | 717 (63%) | 477 (65%) |
| ***Patient Ethnicity*** | Caucasian | 601 | 384 |
|  | Black | 170 | 99 |
|  | Other | 145 | 99 |
|  | Not available^A^ | 364 | 224 |
| ***Primary Disease*** | Diabetic nephropathy | 202 | 144 |
|  | Hypertension / large vessel disease | 107 | 68 |
|  | Glomerulonephritis / vasculitis | 383 | 224 |
|  | Interstitial nephritis / pyelonephritis | 83 | 53 |
|  | Polycystic kidney disease | 122 | 85 |
|  | Others | 128 | 74 |
|  | Unknown etiology | 255 | 158 |
| ***Mean donor age at transplantation (range)*** | | 43 (1 – 85) | 45 (3 – 85) |
| ***Donor gender (% male)*** | | 413 (46%) | 270 (46%) |
| ***Donor type (% deceased donor transplants)*** | | 837 (68%) | 540 (68%) |
| ***Latest kidney status***  ***(% of known)*** | Functioning | 932 (80%) | 630 (85%) |
|  | Failed due to graft failure | 217 (17%) | 101 (12%) |
|  | Failed due to death with function | 15 (1%) | 13 (1%) |
|  | Mean (median) follow-up  (functioning grafts) in days | 775 (465) | 810 (469) |
| ^A^ Some centers elected not to identify ethnicity | | | |

| **Table S3.** Histologic diagnoses and HLA antibody status (All biopsies N=1526) | | | | |
| --- | --- | --- | --- | --- |
| **Characteristics at time of biopsy** | | | **All biopsies**  **(N=1526)** | **No rejection**  **Non-medulla biopsies**  **(N=945)** |
| **Median time of biopsy post-transplant (TxBx) in days (range)** | | | 569 (1 – 12371) | 374 (1 - 11453) |
| **Histology** | | | | |
| **Rejection-related** | **ABMR-related** | ABMR | 289 (19%) | 71 |
|  |  | Transplant glomerulopathy (TG) | 46 (3%) | 16 |
|  |  | ABMR suspected | 29 (2%) | 10 |
|  | Mixed (TCMR plus ABMR) | | 55 (4%) | 5 |
|  | **TCMR-related** | TCMR^A^ | 124 (8%) | 34 |
|  |  | BK virus nephropathy | 45 (3%) | 20 |
| Borderline | | | 120 (8%) | 92 |
| **No rejection** | No major histologic abnormalities ≤6 weeks^B^ | | 112 (7%) | 109 |
|  | Diabetic Nephropathy | | 23 (2%) | 21 |
|  | Glomerulonephritis | | 106 (7%) | 85 |
|  | IFTA not otherwise specified | | 175 (11%) | 145 |
|  | No major histologic abnormalities >6 weeks (NOMOA) | | 333 (22%) | 289 |
|  | Others^C^ | | 69 (5%) | 48 |
| **Patient HLA antibody status** | | | | |
| **DSA at time of biopsy** | | | **All biopsies**  **(N=1526)** | **No rejection**  **Non-medulla biopsies**  **(N=945)** |
| DSA positive | | | 520 (34%) | 250 (26%) |
| DSA negative, PRA positive | | | 215 (14%) | 141 (15%) |
| DSA negative, PRA unknown | | | 118 (8%) | 82 (9%) |
| PRA negative/DSA negative or unknown | | | 435 (29%) | 330 (35%) |
| Unknown DSA/PRA | | | 238 (16%) | 142 (15%) |
| ^A^ Three biopsies had histology diagnoses of both TCMR and BK virus, as characterized previously in Reeve J, Bohmig GA, Eskandary F, Einecke G, Madill-Thomsen K, Mackova M, et al. Generating automated kidney transplant biopsy reports using ensembles of molecular classifiers. Am J Transplant. 2019;19(10):2719-31.  ^B^ Because AKI cannot be diagnosed by histology, this term was used to designate indication biopsies in the first 6 weeks with no evidence of rejection, borderline, or diseases  ^C^ Others includes calcineurin inhibitor toxicity, C4d deposition without morphologic evidence for active rejection, donor origin vascular disease, pyelonephritis, systemic infection/diarrhea, and bacterial infection. | | | | |

| **Table S4.** Mean scores for AKI and CKD-related pathogenesis-based transcript sets (PBTs) in biopsies with no molecular rejection (N=945) | | | | | | | |
| --- | --- | --- | --- | --- | --- | --- | --- |
| **Biological processes** | **Mean transcript set and classifier score^A^ in biopsies grouped by highest archetype score** | **AKI1**  **(N=109)** | **AKI2**  **(N=56)** | **Minor injury**  **(N=185)** | **CKD/AKI**  **(N=7)** | **CKD**  **(N=158)** | **No injury**  **(N=430)** |
| **CKD-related classifiers** | **ci>1_Prob_**^B^  **(interstitial fibrosis)** | 0.21 | 0.29 | 0.30 | **0.75** | 0.74 | *0.17* |
|  | **ct>1_Prob_**^B^  **(tubular atrophy)** | 0.14 | 0.22 | 0.22 | **0.67** | 0.66 | *0.13* |
| **Injury-related classifiers** | **lowGFR_Prob_** | 0.73 | **0.80** | *0.12* | 0.59 | 0.46 | *0.12* |
|  | **Prot_Prob_** | 0.70 | **0.72** | 0.67 | 0.68 | 0.71 | *0.41* |
| **PBTs increased by recent injury** | **AKI transcripts (IRRATs)** | 1.68 | **2.48** | 1.09 | 2.02 | 1.67 | *0.92* |
|  | **IRITD3** | 1.09 | **1.22** | 1.00 | **1.22** | 1.11 | *0.95* |
|  | **IRITD5** | 1.27 | 1.55 | 1.23 | **1.74** | 1.39 | *1.14* |
| **PBTs increased in atrophy-fibrosis** | **IGTs** | *0.87* | 0.96 | 2.21 | 2.79 | **3.02** | 1.30 |
|  | **MCATs** | *1.06* | 1.27 | 2.74 | **6.91** | 5.41 | 1.40 |
|  | **BATs**^B^ | *1.02* | 1.07 | 1.12 | **1.48** | 1.22 | 1.05 |
| **Parenchymal transcript PBTs decreased by injury** | **KT1** | 0.85 | 0.71 | 0.93 | *0.50* | 0.81 | **0.95** |
| **Macrophage infiltration PBTs** | **QCMATs**^B^ | 1.22 | **1.81** | 1.23 | 1.75 | 1.36 | *1.13* |
|  | **AMATs**^B^ | 1.32 | 2.05 | 1.31 | **2.06** | 1.57 | *1.17* |
| **Injury PC1** | | -0.13 | 2.44 | -0.98 | **5.33** | 2.17 | *-2.45* |
| **Injury PC2** | | -1.85 | *-2.70* | 0.63 | 0.09 | **1.40** | -0.11 |
| **Injury PC3** | | **1.27** | 0.19 | 0.34 | *-1.32* | 0.67 | -0.37 |
| The highest in each row is bolded and shaded. The lowest in each row is italicized.  ^A^ The gene sets were derived in human cell lines, human transplants, and mouse models to reflect biological processes relevant to rejection and injury.  ^B^ These were the transcript sets or classifiers not used in the Injury AA analysis.  Abbreviations: AMAT - alternative macrophage associated transcripts 1; BATs – B cell-associated transcripts; DAMPs – damage-associated molecular pattern transcripts; IGTs – immunoglobulin transcripts; IRITD3 - injury-repair induced transcripts day 3; IRITD5 - injury-repair induced transcripts day 5; IRRAT – AKI transcripts; KT1 – kidney parenchymal transcripts 1; KT2 – kidney parenchymal transcripts 2; MCATs – mast cell transcripts; QCMAT - quantitative constitutive macrophage-associated transcripts; | | | | | | | |
